# Supplementary material for: Phylogenetic based dissection of eukaryotic Mo-insertase functionality: From mechanism to complex assembly
Source: PLoS One. 2026 Jun 12;21(6):e0350191. doi: 10.1371/journal.pone.0350191 (PMC13262936; doi:10.1371/journal.pone.0350191)
Supplement: S1 Fig — Species name and accession number of the identified MoeA homologous sequence are given next to the branches. *The eukaryotic E-domain sequence XP 682307.1 from Aspergillus nidulans was identified to group better with prokaryotic than eukaryotic (fungal) sequences. (PDF) [file pone.0350191.s001.pdf]

Prokaryotes

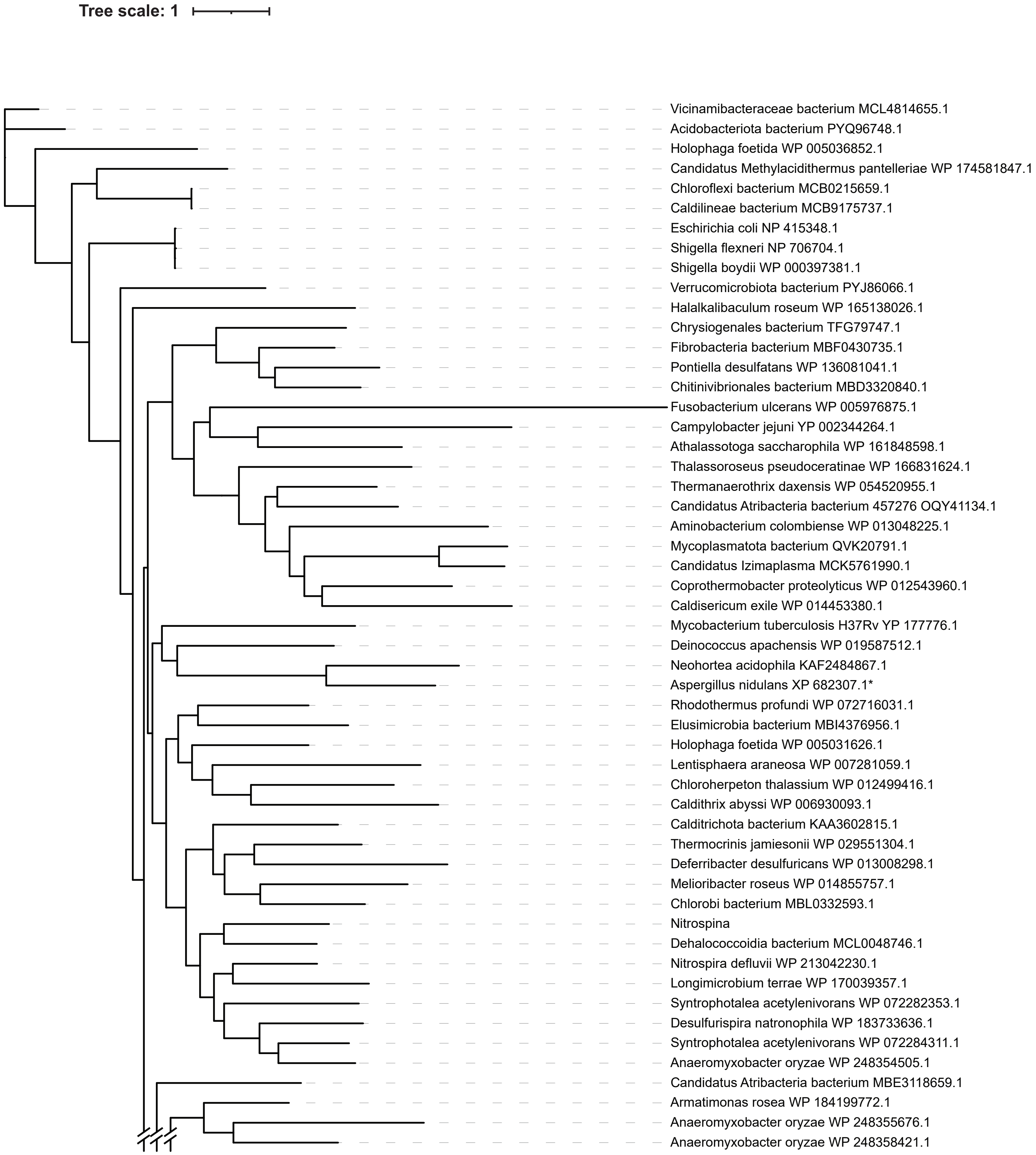

Figure S1: Partial representation of the phylogenetic distance tree obtained from maximum likelihood: Prokaryotes. Species name and accession number of the identified MoeA homologous sequence are given next to the branches. *The eukaryotic E-domain sequence XP 682307.1 from *Aspergillus nidulans* was identified to group better with prokaryotic than eukaryotic (fungal) sequence.
