# Supplementary material for: Phylogenetic based dissection of eukaryotic Mo-insertase functionality: From mechanism to complex assembly
Source: PLoS One. 2026 Jun 12;21(6):e0350191. doi: 10.1371/journal.pone.0350191 (PMC13262936; doi:10.1371/journal.pone.0350191)
Supplement: S2 Fig — Species name and the accession number of the identified MoeA homologous sequence are given next to the branches. *The algal E-domain sequence KAG5179608.1 from Tribonema minus was identified to group better with fungal than plant sequences. (PDF) [file pone.0350191.s002.pdf]

Fungi

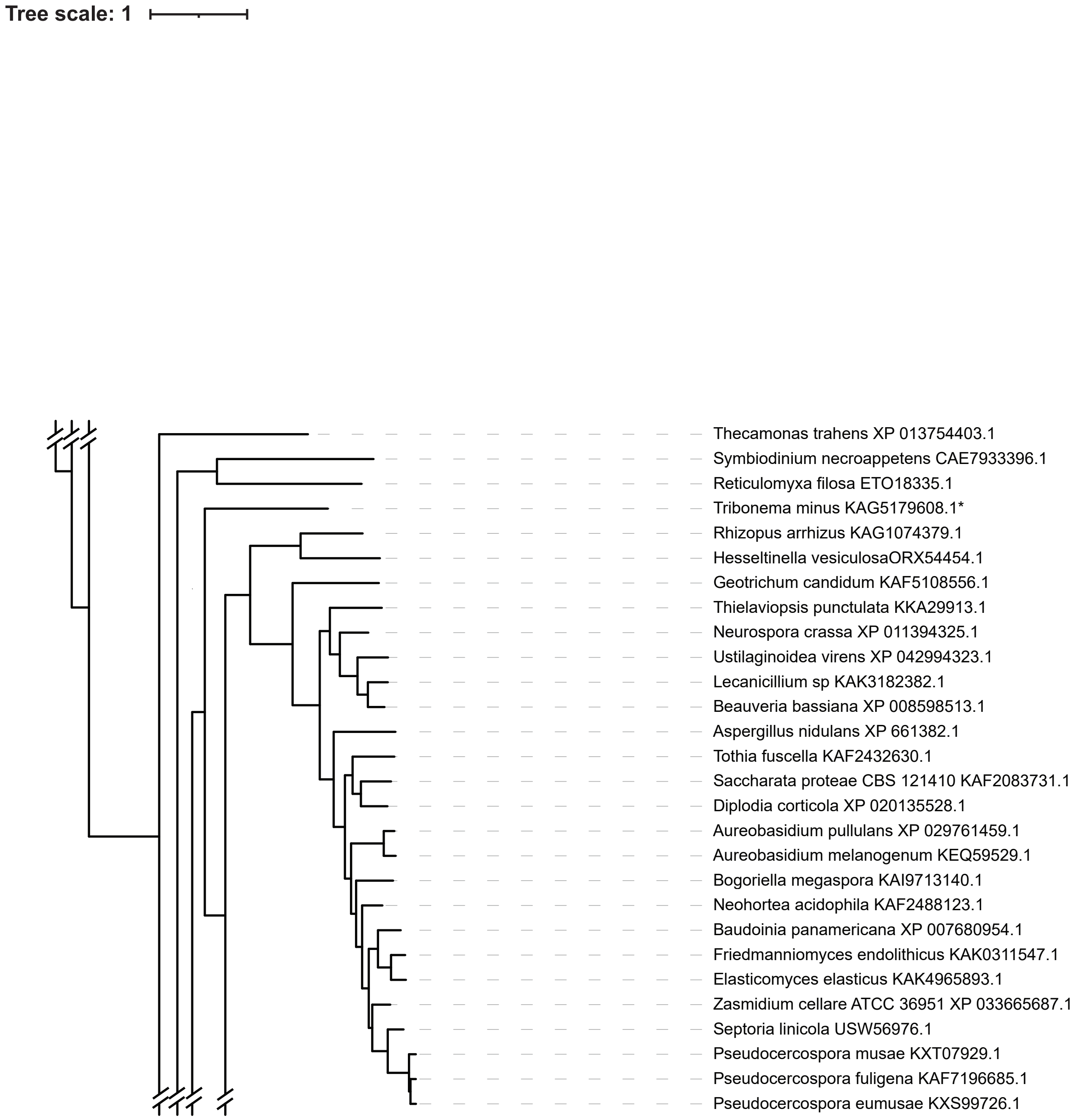

Figure S2: Partial representation of the phylogenetic distance tree obtained from maximum likelihood: Fungi. Species name and the accession number of the identified MoeA homologous sequence are given next to the branches. *The algal E-domain sequence KAG5179608.1 from *Tribonema minus* was identified to group better with fungal than plant sequences.
