# Supplementary material for: Phylogenetic based dissection of eukaryotic Mo-insertase functionality: From mechanism to complex assembly
Source: PLoS One. 2026 Jun 12;21(6):e0350191. doi: 10.1371/journal.pone.0350191 (PMC13262936; doi:10.1371/journal.pone.0350191)
Supplement: S4 Fig — Species name and the accession number of the identified MoeA homologous sequence are given next to the branches. (PDF) [file pone.0350191.s004.pdf]

Plants I

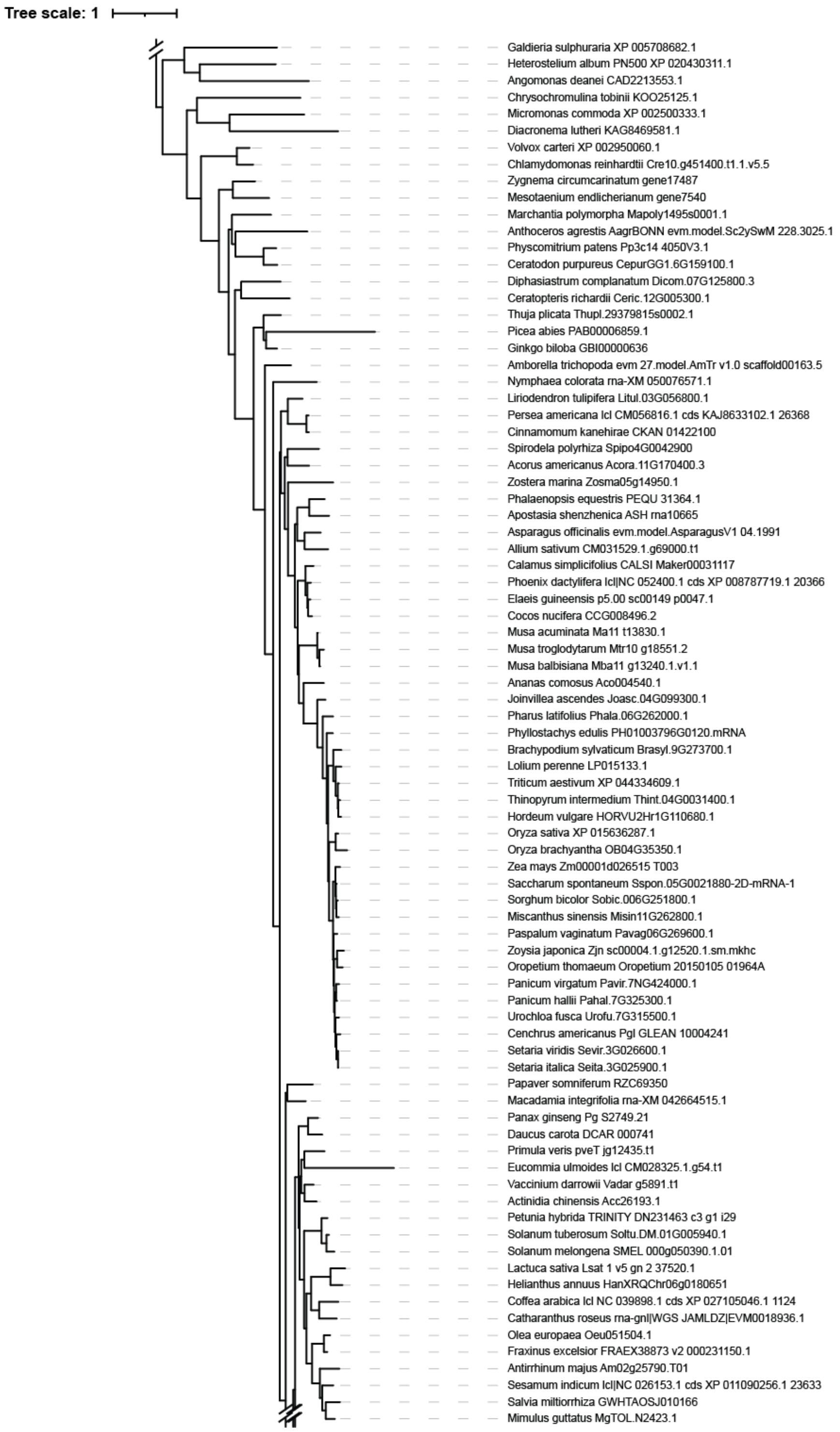

Figure S4: Partial representation of the phylogenetic distance tree obtained from maximum likelihood: Plants I of II. Species name and the accession number of the identified MoeA homologous sequence are given next to the branches.
