# Supplementary material for: Phylogenetic based dissection of eukaryotic Mo-insertase functionality: From mechanism to complex assembly
Source: PLoS One. 2026 Jun 12;21(6):e0350191. doi: 10.1371/journal.pone.0350191 (PMC13262936; doi:10.1371/journal.pone.0350191)
Supplement: S6 Fig — Mo-insertase domains identified in A. nidulans (A) and G. candidum (B). (A) and (B): domains were annotated according to A. nidulans CNXE domain annotation (Probst, C., et al., Genetic characterization of the Neurospora crassa molybdenum cofactor biosynthesis. Fungal Genet Biol, 2014. 66: p. 69–78.). Our initial BLASTp approach (see the materials and methods section for details) identified proteins XP682307.1 and XP661382 (A. nidulans) and KAF5108556 (G. candidum). (A) the A. nidulans Mo-insertase full length Mo-insertase domain organization is shown according to (Probst, C., et al., Genetic characterization of the Neurospora crassa molybdenum cofactor biosynthesis. Fungal Genet Biol, 2014. 66: p. 69–78.). The schematic domain organization shown was taken from Fig 3. (B) the G. candidum full length Mo-insertase (CDO51814.1) was identified by an BLASTp search using the NCBI protein database (non-redundant protein sequences (nr), default settings) in the taxon G. candidum and using full length A. nidulans Mo-insertase (AAK83300.1) as query. The sequence CDO51814.1 is not included in the phylogenetic trees constructed within this work. (PDF) [file pone.0350191.s006.pdf]

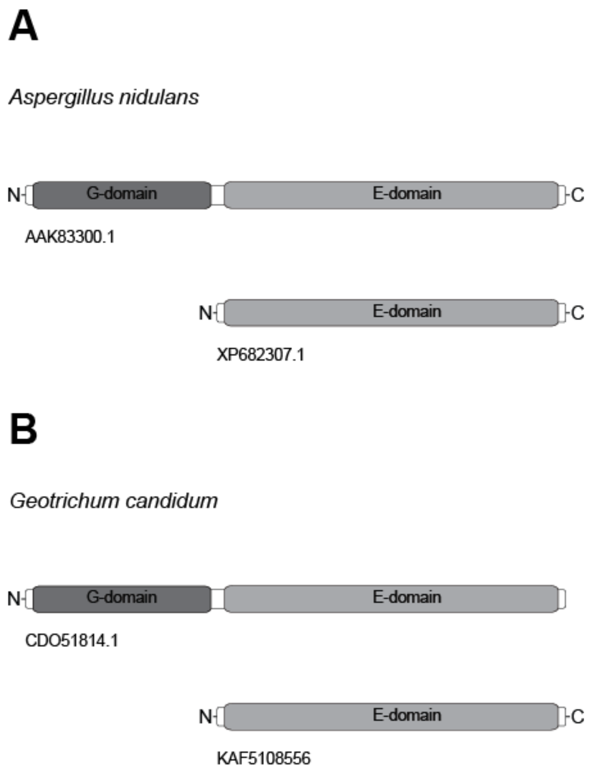

Figure S6: Schematic representation of identified Mo-insertase domains in *Aspergillus nidulans* and *Geotrichum candidum*. Mo-insertase domains identified in *A. nidulans* (A) and *G. candidum* (B). (A) and (B): domains were annotated according to *A. nidulans* CNXE domain annotation (Probst, C., et al., Genetic characterization of the Neurospora crassa molybdenum cofactor biosynthesis. Fungal Genet Biol, 2014. **66**: p. 69-78.). Our initial BLASTp approach (see the materials and methods section for details) identified proteins XP682307.1 and XP661382 (*A. nidulans*) and KAF5108556 (*G. candidum*). (A) the *A. nidulans* Mo-insertase full length Mo-insertase domain organization is shown according to (Probst, C., *et al.*, Genetic characterization of the *Neurospora crassa* molybdenum cofactor biosynthesis. Fungal Genet Biol, 2014. **66**: p. 69-78.). The schematic domain organization shown was taken from Fig. 3. (B) the *G. candidum* full length Mo-insertase (CDO51814.1) was identified by an BLASTp search using the NCBI protein database (non-redundant protein sequences (nr), default settings) in the *taxon G. candidum* and using full length *A. nidulans* Mo-insertase (AAK83300.1) as query. The sequence CDO51814.1 is not included in the phylogenetic trees constructed within this work.
