# Supplementary material for: Phylogenetic based dissection of eukaryotic Mo-insertase functionality: From mechanism to complex assembly
Source: PLoS One. 2026 Jun 12;21(6):e0350191. doi: 10.1371/journal.pone.0350191 (PMC13262936; doi:10.1371/journal.pone.0350191)
Supplement: S7 Fig — Patristic distance of MOCS2B and alcohol dehydrogenase from indicated taxons. For comparison the patristic distance determined for gephyrin (GEPH) in the taxon Gnathostomata is given. The estimated age when the compared taxons emerged is indicated (MYA = million years ago). For calculation of patristic distances, the taxons Amphibia (320 MYA) and Sauria (280 MYA) were combined. Estimates of taxon ages were extracted from the evolutionary time tree of life (Kumar, S., et al., TimeTree 5: An Expanded Resource for Species Divergence Times. Mol Biol Evol, 2022. 39(8) and Kumar, S., et al., TimeTree: A Resource for Timelines, Timetrees, and Divergence Times. Mol Biol Evol, 2017. 34(7): p. 1812–1819.). (PDF) [file pone.0350191.s007.pdf]

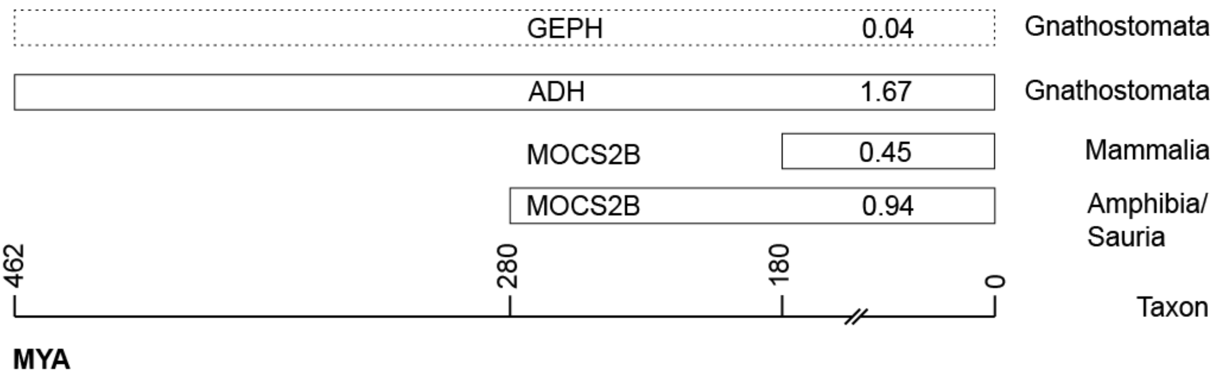

Figure S7: Patristic distance of the MOCS2B and alcohol dehydrogenase from different taxons. Patristic distance of MOCS2B and alcohol dehydrogenase from indicated taxons. For comparison the patristic distance determined for gephyrin (GEPH) in the taxon *Gnathostomata* is given. The estimated age when the compared taxons emerged is indicated (MYA = million years ago). For calculation of patristic distances, the taxons *Amphibia* (320 MYA) and *Sauria* (280 MYA) were combined. Estimates of taxon ages were extracted from the evolutionary time tree of life (Kumar, S., *et al.*, TimeTree 5: An Expanded Resource for Species Divergence Times. Mol Biol Evol, 2022. **39**(8) and Kumar, S., *et al.*, TimeTree: A Resource for Timelines, Timetrees, and Divergence Times. Mol Biol Evol, 2017. 34(7): p. 1812-1819.)
