# Supplementary material for: Phylogenetic based dissection of eukaryotic Mo-insertase functionality: From mechanism to complex assembly
Source: PLoS One. 2026 Jun 12;21(6):e0350191. doi: 10.1371/journal.pone.0350191 (PMC13262936; doi:10.1371/journal.pone.0350191)
Supplement: S8 Fig — Surface representations of the R. norvegicus (PDB code: 2FU3, A and B) and A. thaliana (PDB code: 6Q32, C) Mo-insertase E-domain. Colors indicate the degree of conservation of surface exposed amino acids amongst members of the Invertebrate-type Mo-insertase (A) the Gnathostome-type Mo-insertase (B) and the Plant-type Mo-insertase (C). The color of the protein surface correlates with the degree of conservation as indicated. The active site as identified for the plant-type Mo-insertase Cnx1 (Probst, C., et al., Mechanism of molybdate insertion into pterin-based molybdenum cofactors. Nat Chem, 2021. 13(8): p. 758–765.) is encircled. (PDF) [file pone.0350191.s008.pdf]

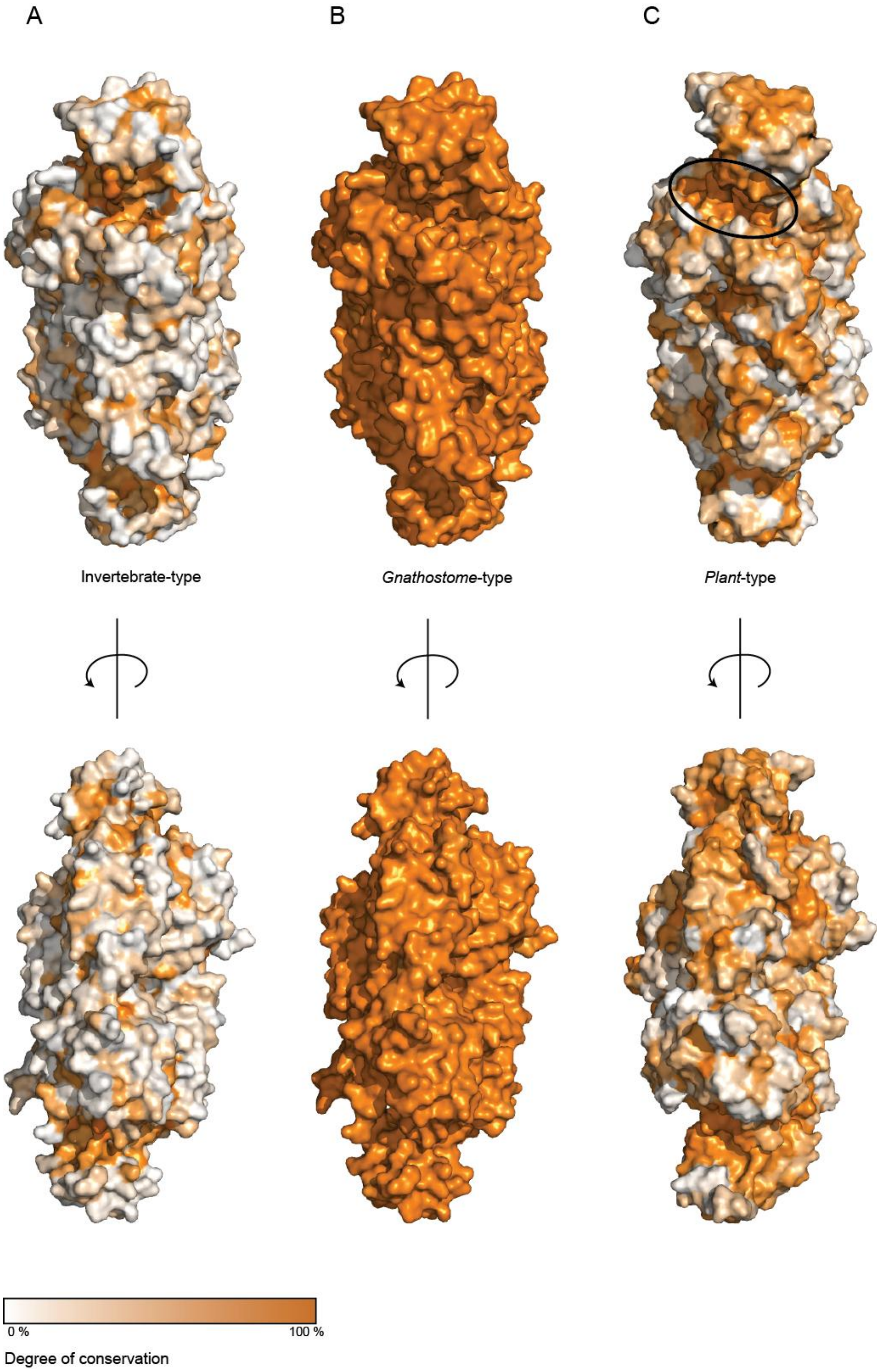

Figure S8: Conserved residues of eukaryotic Mo-insertases. Surface representations of the *R. norvegicus* (PDB code: 2FU3, A and B) and *A. thaliana* (PDB code: 6Q32, C) Mo-insertase E-domain. Colors indicate the degree of conservation of surface exposed amino acids amongst members of the Invertebrate-type Mo-insertase (A) the Gnathostome-type Mo-insertase (B) and the Plant-type Mo-insertase (C). The color of the protein surface correlates with the degree of conservation as indicated. The active site as identified for the plant-type Mo-insertase Cnx1 (Probst, C., *et al.*, Mechanism of molybdate insertion into pterin-based molybdenum cofactors. Nat Chem, 2021. **13**(8): p. 758-765.) is encircled.
