# Supplementary material for: Phylogenetic based dissection of eukaryotic Mo-insertase functionality: From mechanism to complex assembly
Source: PLoS One. 2026 Jun 12;21(6):e0350191. doi: 10.1371/journal.pone.0350191 (PMC13262936; doi:10.1371/journal.pone.0350191)
Supplement: S9 Fig — Partial representation of modelled variants (blue) superimposed with the wildtype Cnx1E structure (6ETF; Krausze, J., et al., The functional principle of eukaryotic molybdenum insertases. Biochem J, 2018. 475(10): p. 1739–1753., grey). RMSD = root mean square deviation. (PDF) [file pone.0350191.s009.pdf]

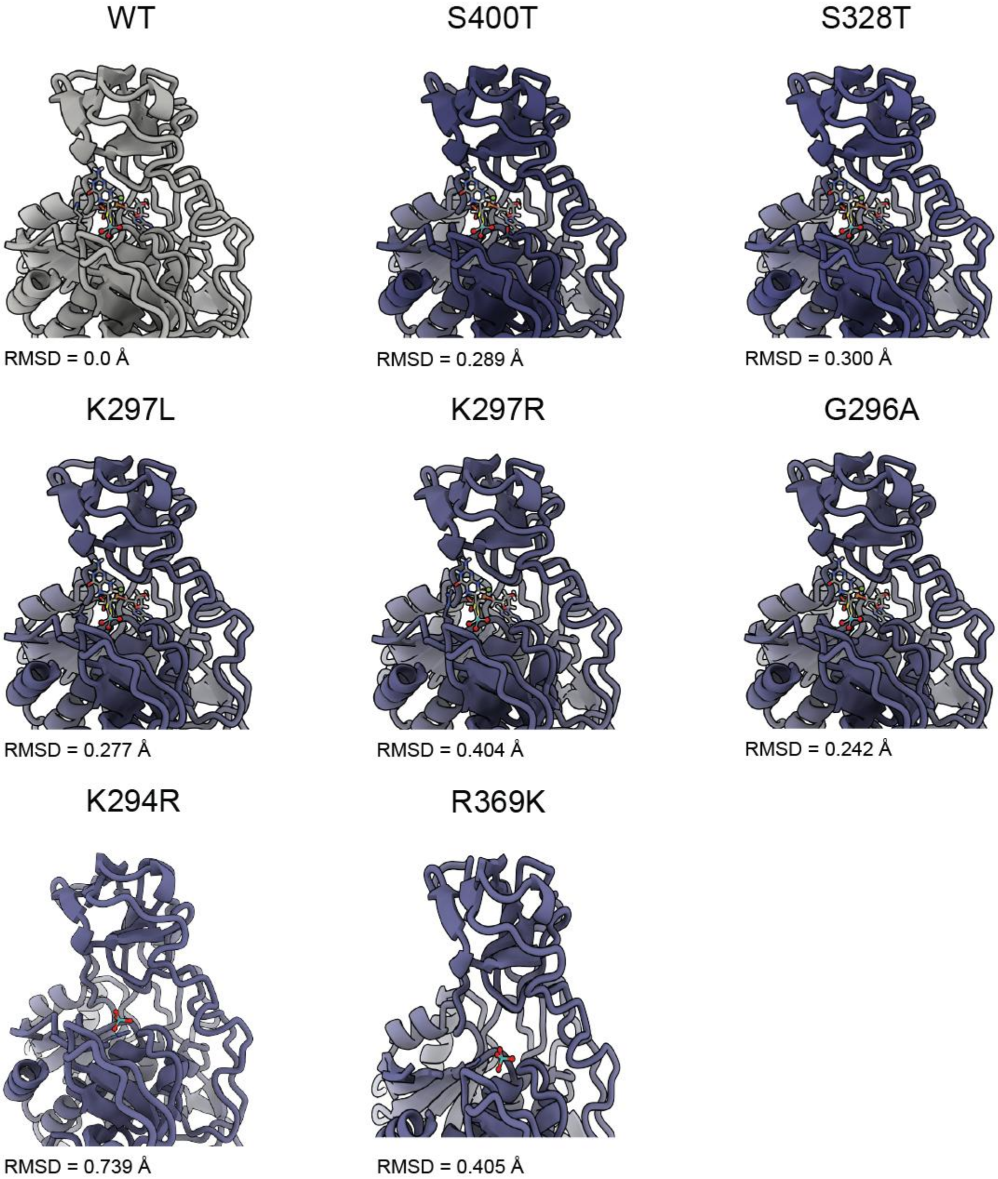

Figure S9: AlphaFold-Based modelling of Mo-insertase active site variants. Partial representation of modelled variants (blue) superimposed with the wildtype Cnx1E structure (6ETF; Krausze, J., *et al.*, The functional principle of eukaryotic molybdenum insertases. Biochem J, 2018. **475**(10): p. 1739-1753., grey), RMSD = root mean square deviation.
