# Supplementary material for: Phylogenetic based dissection of eukaryotic Mo-insertase functionality: From mechanism to complex assembly
Source: PLoS One. 2026 Jun 12;21(6):e0350191. doi: 10.1371/journal.pone.0350191 (PMC13262936; doi:10.1371/journal.pone.0350191)
Supplement: S10 Fig — Surface representations of the R. norvegicus (PDB code: 2FU3) Mo-insertase E-domain. Highly conserved (> 70% identity) residues of Invertebrate-type Mo-insertases are shown color coded as specified in S8 Fig. Residues that fall below this threshold are shown in grey. (The receptor binding site (according to Maric, H.M., et al., Gephyrin-mediated gamma-aminobutyric acid type A and glycine receptor clustering relies on a common binding site. J Biol Chem, 2011. 286(49): p. 42105–42114.) is encircled. (PDF) [file pone.0350191.s010.pdf]

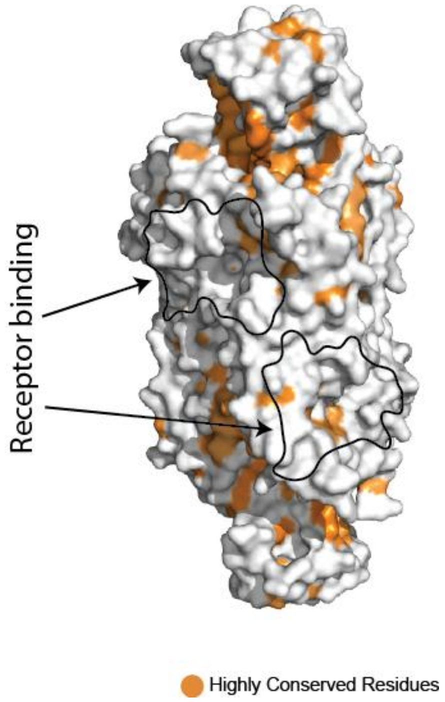

Figure S10: Highly conserved residues of Invertebrate-type Mo-insertase. Surface representations of the *R. norvegicus* (PDB code: 2FU3) Mo-insertase E-domain. Highly conserved (> 70% identity) residues of Invertebrate-type Mo-insertases are shown color coded as specified in Fig. S8. Residues that fall below this threshold are shown in grey. (The receptor binding site (according to Maric, H.M., et al., Gephyrin-mediated gamma-aminobutyric acid type A and glycine receptor clustering relies on a common binding site. J Biol Chem, 2011. 286(49): p. 42105-42114.) is encircled.
