# Supplementary material for: Phylogenetic based dissection of eukaryotic Mo-insertase functionality: From mechanism to complex assembly
Source: PLoS One. 2026 Jun 12;21(6):e0350191. doi: 10.1371/journal.pone.0350191 (PMC13262936; doi:10.1371/journal.pone.0350191)
Supplement: S1 Table — Mo-insertases possessing a diverging domain arrangement as compared to the clade (fungi, animals, plants) where these grouped to (see Fig 3 for comparison) are tabulated. The E-domains tabulated were identified by the initial BLASTp search. If indicated (i.e., when a separate existent E-domain was identified, G-domains were identified by using the MogA sequence (QKU47929.1) as query for a BLASTp search and restricted to the respective organism, using the NCBI protein database (non-redundant protein sequences (nr), default settings). As an exception for the identification of the Volvox carteri G-domain BLASTp searches (standard settings) were carried out using the JGI database (Grigoriev, I.V., et al., The genome portal of the Department of Energy Joint Genome Institute. Nucleic Acids Res, 2012. 40 (Database issue): p. D26-32) with queries restricted to Volvox carteri. If indicated the number of the first and last amino acid of the E- and G-domain within the fusion proteins are given. For Tribonema minus, Symbiodinium necroappetens and Heterostelium album the structure of the G-domain has been predicted using (Powell, H.R., et al., Phyre2.2: A Community Resource for Template-based Protein Structure Prediction. J Mol Biol, 2025. 437(15): p. 168960. to refine domain annotation.) For Reticulomyxa filose (ETO18335.1) only a partial sequence was available which showed significant sequence similarities to the H. sapiens gephyrin E-domain. The Pinctada imbricata G-domain is part of a hypothetical protein comprising 1183 aa. In Diacronema lutheri, the E-domain was identified to be part of a hypothetical protein comprising 611 aa (KAG8469581.1) respectively. The G-domains identified in Micromonas commode, Diacronema lutheri and Chrysochromulina tobinii possessed an N-terminal extension comprising ca. 150 residues. Domain classification in fusion proteins was carried out as described in the materials and methods section. The C. reinhardtii Mo-insertase domain organization was [file pone.0350191.s011.pdf]

Table S1: Mo-insertases with a diverging domain arrangement. Mo-insertases possessing a diverging domain arrangement as compared to the clade (fungi, animals, plants) where these grouped to (see Fig. 3 for comparison) are tabulated. The E-domains tabulated were identified by the initial BLASTp search. If indicated (*i.e.* when a separate existent E-domain was identified, G-domains were identified by using the MogA sequence (QKU47929.1) as query for a BLASTp search and restricted to the respective organism, using the NCBI protein database (non-redundant protein sequences (nr), default settings). As an exception for the identification of the *Volvox carteri* G-domain BLASTp searches (standard settings) were carried out using the JGI database (Grigoriev, I.V., *et al.*, The genome portal of the Department of Energy Joint Genome Institute. Nucleic Acids Res, 2012. **40**(Database issue): p. D26-32) with queries restricted to *Volvox carteri*. If indicated the number of the first and last amino acid of the E- and G-domain within the fusion proteins are given. For *Tribonema minus*, *Symbiodinium necroappetens* and *Heterostelium album* the structure of the G-domain has been predicted using (Powell, H.R., et al., Phyre2.2: A Community Resource for Template-based Protein Structure Prediction. J Mol Biol, 2025. 437(15): p. 168960. to refine domain annotation.) For *Reticulomyxa filose* (ETO18335.1) only a partial sequence was available which showed significant sequence similarities to the *H. sapiens* gephyrin E-domain. The *Pinctada imbricata* G-domain is part of a hypothetical protein comprising 1183 aa. In *Diacronema lutheri*, the E-domain was identified to be part of a hypothetical protein comprising 611 aa (KAG8469581.1) respectively. The G-domains identified in *Micromonas commode*, *Diacronema lutheri* and *Chrysochromulina tobinii* possessed an N-terminal extension comprising ca. 150 residues. Domain classification in fusion proteins was carried out as described in the materials and methods section. The *C. reinhardtii* Mo-insertase domain organization was described elsewhere (Llamas, A., et al., Molybdenum metabolism in the alga Chlamydomonas stands at the crossroad of those in Arabidopsis and humans. Metallomics, 2011. 3(6): p. 578-90.). For Streptophyta species where separate E-domains were identified, G-domain containing sequences were identified by using the *A. thaliana* Cnx1G sequence (Krausze, J., *et al.*, Dimerization of the plant molybdenum insertase Cnx1E is required for synthesis of the molybdenum cofactor. Biochem J, 2017. 474(1): p. 163-178. and reference therein) as query for a BLASTp searches and restricted to the respective organism, using the NCBI protein database (non-redundant protein sequences (nr), default settings). The *Carya illinoinensis*, *Tripterygium wilfordii* and *Nymphaea colorata* G-domain containing sequences (KAG2674269.1, XP_038715653.1 and XP_031481496.1 respectively) comprises G- and E-domains with the plant type orientation, indicated by an asterisk.

Clade	Species	E-domain	G-domain
**Fungi**	*Tribonema minus*	KAG5179608.1	
		(16-448)	(480-620)
	Symbiodinium necroappetens	CAE7933396.1	
		(10-439)	(468-608)
	Reticulomyxa filose	ETO18335.1	
		(partial)	n.a.
**Animals**	*Trichuris trichiura*	CDW51849.1	CDW57105.1
	Caenorhabditis elegans	NP_509700.2	NP_001370857.1
	Pinctada imbricata	KAK3092834.1	KAK3093217.1
	Ephemera danica	KAF4523361.1	n.a.
**Plants**	*Carya illinoinensis*	CiLak.13G125300.1	KAG2674269.1*
	Eucommia ulmoides	lcl_CM028325.1.lcl _CM028325.1.g54.t1	n.a.
	Fagus sylvatica	FSB015820001	n.a.
	Gynostemma penaphyllum	CM035884.1. g4717.t1	n.a.
	Luffa aegyptiaca	CM029397.1 g6424.t1	n.a.
	Nymphaea colorata	XM_050076571.1	XP_031481496.1*
	Tripterygium wilfordii	NC_05223.1_cds XP_038715662.1 _2868	XP_038715653.1*
	Solanum melongena	SMEL_000g050 390.1.01	n.a.
	Micromonas commoda	XP_002500333.1	XP_002504805.1
	Volvox carteri	XP_002950060	Vocar.0002s0019.1.p
	Chlamydomonas reinhardtii	DQ311646.1	DQ311645.1
	Galdieria sulphuraria	XP_005708682.1	n.a.
	Angomonas deanei	CAD2213553.1	n.a.
	Diacronema lutheri	KAG8469581.1	KAG8466760.1
	Chrysochromulina tobinii	KOO25125.1	KOO34835.1
	Heterostelium album	XP_020430311.1	
		(245-671)	(11-181)
