# Supplementary material for: Phylogenetic based dissection of eukaryotic Mo-insertase functionality: From mechanism to complex assembly
Source: PLoS One. 2026 Jun 12;21(6):e0350191. doi: 10.1371/journal.pone.0350191 (PMC13262936; doi:10.1371/journal.pone.0350191)
Supplement: S2 Table — Contaminated sequences and G-domain like sequences identified within the dataset are tabulated. Databases: Pucker et al., 2024 (Pucker, B., Fiene, N., Choudhary, N., Borchert, M., Khatun, N., Collection of plant gene expression data. https://doi.org/10.24355/dbbs.084-202409160820-0. 2024.); O’Leary et al., 2024 (O’Leary, N.A., et al., Exploring and retrieving sequence and metadata for species across the tree of life with NCBI Datasets. Sci Data, 2024. 11(1): p. 732.). (PDF) [file pone.0350191.s012.pdf]

Table S2: Sequences not considered for analysis. Contaminated sequences and G-domain like sequences identified within the dataset are tabulated. Databases: Pucker *et al.*, 2024 (Pucker, B., Fiene, N., Choudhary, N., Borchert, M., Khatun, N., Collection of plant gene expression data. https://doi.org/10.24355/dbbs.084-202409160820-0. 2024.); O'Leary *et al.*, 2024 (O'Leary, N.A., *et al.*, Exploring and retrieving sequence and metadata for species across the tree of life with NCBI Datasets. Sci Data, 2024. 11(1): p. 732.).

**Originally identified in Species / Database**	**Sequence**	**Reason for elimination**
Tamarix ramosissima /<br>Pucker *et al.*, 2024	AKPGTMASSTPQSLLRAAILIVSDTASKDPSTDKAAPVLKEV<br>FETEGAGKWDTATAGSSDSNGIAVKIVPDHKAQIQDAITTWA<br>DSEDFYNLIITTGGTGFTPKDNTPEAVSPLLHRHAPGLVHGI<br>LAASFEVTPFAMMSRPVAGVRNKTIIITLPGSPKGAKENLQA<br>VLKLLPHACLQAAGENSRAAHAGGVKQLEKDAGVASFSVKGK<br>TAHAHNRDLGHSHSHDHSHGHGHGGHAVPKAHTSPSQRPPQS<br>NDPRLGASHRARQSPYPMLSVSEAVDTILSHTPSPGPTTAPL<br>SISLVGAVISSDIKAPEAVPAYRASIVDGYAIIVPEDPSQRH<br>TTKGTFPVASVSHAQASSMPPPLQSGEIARITTGAPLPDNAN<br>AVVMVEDTVIASLTSTDPAEEKEVTILTDALVPGENIREPGS<br>DIALNSTILTSGARITGLGGEIGLLAASGTHTVPVYRRPRVG<br>VLSTGDEVTDISHPGPLTGGMIRDSNRPSLLSLISSWRLCSE<br>VVDLGIARDTPPSDLETKLRDAYRVQDLDVVVTTGGVSMGEL<br>DLLKPTIERTLGGTIHFGRVNMKPGKPTTFGSVPVKTNDGTQ<br>RNERLIFALPGNPASALVTANLFLLPALQKLAGIKDVTGLER<br>VDVRLAGRVRCDKSRPEYHRCTVHFDGTSGGLVAVSTGMQRS<br>SRVGSVGGANGLLCLPVKEGHLEEGEKCECLLMGMIVGS	**contaminated sequence**<br><br>**correct assignment:**<br>*Knufia fluminis*
Tamarix ramosissima /<br>Pucker *et al.*, 2024	FRKESKWPMIPIDEALQIVLNQTEILPAKKVALKNSLGHIVA<br>EDILAKEPLPPFRASIKDGFAVRFQDGPGVYPVIGTVTAGVI<br>PNFKVESKTIARITTGSALPEGADAIVMVERTELLPDRDEQG<br>RELVRILEGAGEGHDIRMIGSDVAIGELVLKKGERIGPAEVG<br>LLATVGVSDVPVIQSPKIAVLSTGDDLCETDQPLTPGHIRDS<br>NRSMLIAAIRESEVSWNESCID	**contaminated sequence**<br><br>**correct assignment:**<br>*Litorilinea sp.*
Idotea baltica /<br>O'Leary *et al.*, 2024	MDCCATQGLIPVETALDTLLSQVSPISNTVVLPLSDAIGFVL<br>ADDICSPINVPPFANSAMDGYAVRISDLEQSLTLPLAGKSFA<br>GIPFDGEWATQTTIRIMTGAKIPTGCDAVIMQELTSDSDGNI<br>TFDLSLTDVKPQTNIRPIGDDVAQGQTVLEKGHRLTPRDIPL<br>IASLGINDIPVVAKPKVAFFSTGDELKPLGQPLEDGQIYDSN<br>RYGIKVLIERFGCEAIDLGIIPDCPETLRKVFLKADKEADVV<br>VTSGGVSVGEADYTKDILDELGQIGFWKLAIKPGKPFAFGNL<br>PNSYFCGLPGNPVSAMLTMYVLVQPMLAKLAGHSSWEAPKSI<br>PAVATTLFKKRPGRTDYQRGIYSINAQGQFEVATTGNQGSGA<br>FSSMSIANCFVVLERERGRVESGEMVNIELFNSSLY	**contaminated sequence**<br><br>**correct assignment:**<br>*Aliivibrio fischeri*
Tamarix ramosissima /<br>Pucker *et al.*, 2024	SGGVSMGDRDFVKPLLEEKGKVYFSKVLMKPGKPLTFAEIRA<br>KPTESMLGKTVLAFGLPGNPVSCLVCFNIFVVPTIRQLAGWT<br>SPHPLRVRLRLQEPIKSDPIRPEFHRAIIKWKTTMDQGLPDL<br>LLRALDIR	**contaminated sequence**<br><br>**correct assignment:**<br>*Arabidopsis thaliana*

**Originally identified in Species / Database**	**Sequence**	**Reason for elimination**
Tamarix ramosissima / Pucker *et al.*, 2024	AIQLATGVNTHSHPSNTKSGCRCSDTTINNSKYEMIEYDVAL<br>QTVLDQSSAVTPDTETLLTTESLGRVTASNIHSTVNIPSYNT<br>SYVDGYALSSITTTNTFTVLTSYTAGDTLISSINIGDGECVR<br>VNTGSLIPINTRYIVMVEDTSLSGDTTITLSTAVTAESMEGN<br>IRRIGTDLRIGDLLLHSNHTITPFDIPLLVSAGIHTITCYKQ<br>CSIGILSSGNEVVDISSTNNTTTTTVVDGMIYDTNRPALIA<br>LFKSHGVNIVDFGIVRDERQEIVHRMTESLKQVDVLITTGGV<br>SMGEKDYIKPILEQEMNSTIHFGRVNLKPGKPTTFATLTLPD<br>DKKKKKFVFGLPGNPVSAIVTSILFVLPLARKMSGHAGYLNE<br>AIPVVIGNDIHLDSRREFMRATVTKEVIGNGGSVRYVATSCG<br>KQTSSRLLSMKGADVLLKLPPREKVGGDGILKKGSVVDAILL<br>K	**contaminated sequence**<br><br>**correct assignment:** *Basidiobolus meristosporus CBS 931.73*
Cullicoides impunctatus / O'Leary *et al.*, 2024	MEPFTQGLIALDDALKVMLESITPLTDDELSMLREASGRITA<br>TAIISPVDVPPFANAAMDGYALRYADFATDRIFPVAGKALAG<br>FPFSEPWPTGSCVRIMTGAPLPAGADVVIMQELATVEGDGVR<br>FNQPVSPGQHIRLAGEDICAGSAIIEPGRRLGTAQLPLCASL<br>GLNQLSVIRRPKVALFSTGDELQLPGQPLSEGQIYDTNRLAV<br>GIMLEKLGCEVRDFGIIPDCPETLKHTFTEADSWADVTISSG<br>GVSVGEADYTRTILESLGKITFWKLAIKPGKPFAFGRLQHSW<br>FCGLPGNPVSAVVSFYQLVQPLLRHLAGEKNITPPRLRARLD<br>GRIKRNPGRIDFQRGRLYQSPEGQFRVTTTGAQGSHVFSSFN<br>QANCFIVLPREQGDVSPDEWVEAEPFNHLLQG	**contaminated sequence**<br><br>**correct assignment:** *Candidatus Erwinia impunctatus*
Ephemera Danica / O'Leary *et al.*, 2024	MTDNSSCISCATENNLLSVAEAREHMIAEVQSITGREFLSLR<br>NALGRVLATDIIAPHDVPAHDNSAMDGYAVCFDSLAAEGETR<br>LTVVGTAFAGNAFSGQVGRGQAVRIMTGAVLPAGADTVVVQE<br>VVRREGSEVVVPAGQQVGQNTRRAGEDLARGAVALPAGKRIG<br>PAELGLVASLGVAEVAVKRRLRVAFFSTGDELASIGKPLAPG<br>EVYDSNRYTLHGLLTRLGAEIIDLGVVPDRPEALEAALAEAA<br>QIADAIITTGGVSVGEADFVREILDKLGEVRFWKINIKPGRP<br>MAFGKVGKAWLFGLPGNPVAVMVSYTQLALGALYRLSGLDPL<br>PERPLLAAISANPIRKQAGRREYLRGRIAAVDGAWQVKTAGN<br>QGSGVLRSMSEANCFVVLPEDCTSVASGDPVAVELFDGLF	**contaminated sequence**<br><br>**correct assignment:** *Dechloromonas sp.*
Trichuris trichiura / O'Leary *et al.*, 2024	MEFTTGLMSLDTALNEMLSRVTPLTPQETLPLVQCFGRILAS<br>DVVSPLDVPGFDNSAMDGYAVRLADIASGQPLPVVGKSFAGQ<br>PYHGEWPAGTCIRIMTGAPVPEGCEAVVMQEQTEQTDNGVRF<br>TAEVRSGQNIRRRGEDISAGAVVFPAGTRLTTAELPVIASLG<br>IAEVPVIRKVRVALFSTGDELQLPGQPLGDGQIYDTNRLAVH<br>LMLEQLGCEVINLGIIRDDPHALRAAFIEADSQADVVISSGG<br>VSVGEADYTKTILEELGEIAFWKLAIKPGKPFAFGKLSNSWF<br>CGLPGNPVSATLTFYQLVQPLLAKLSGNTASGLPARQRVRTA<br>SRLKKTPGRLDFQRGVLHRNADGELEVTTTGHQGSHIFSSFS<br>LGNCFIVLERDRGNVEVGEWVEVEPFNALFGGL	**contaminated sequence**<br><br>**correct assignment:** *Escherichia coli*
Russula earlei / O'Leary *et al.*, 2024	MITVNEAKNIIRHNCKVLPAITLPLESALTYVLAEDVYAVAD<br>IPAFDQSSMDGYAIAFDDYYYHHKLQVEGVIPAGHSIAARIQ<br>ARQAARIFTGAPMPQGADTVIIQEKVTVENNELTGTDTLLKK<br>GSNVRPKGSEIKAGEIAATKGTCLSPAAIGFLAGIGIAEVKV<br>ISKPTISIVVTGNELRQPGKPLLHGQVYESNSFTLNAILQQL<br>FMSNVTTIWVDDDLCLMEDALEKALEHSDMVLLTGGVSVGDY<br>DYVLEAASICGVQQLFHRIKQKPGKPLFFGKKGDKLVFGLPG<br>NPSSVLTCFYEYVIPALQQLTQRKSIIKVVHLPLAKAHQKKP<br>GLTHFLKGHVEANKVIPLQAQESYRLSSFSAANCLIRLEEEG<br>EEYAAGAMVEVHLLPF	**contaminated sequence**<br><br>**correct assignment:** *Flavisolibacter sp.*

**Originally identified in Species / Database**	**Sequence**	**Reason for elimination**
Symbiodinium necroappetens / O'Leary *et al.*, 2024	AFLVGESLMRQAEVTAATRALLAGKAVMVDVGAKAETERRAT<br>ARGEVVMQPETLRLIESGGVQKGDVLSVARLAGIMGAKRTPE<br>LIPLCHPLALTSVTLDLRLRPERDAVEIEATCKLTGRTGVEM<br>EALTAVSVAALTVYDMCKAVDRGMRIDNVRLVHKTGGKSGTY<br>EDDEAECFFATARPRDARPGDREPPQRSSGMISVEEAQTRVL<br>AAFSPLPAETVAVNQALGRVLAEDVTARVTQPPADVSAMDGY<br>AVRAEDLAEIPARLQVVGRVPAGGRYADRLAPGQAVRIFTGA<br>PLPDGADTIVIQEDCTAEGDAVVVRAGAARGTYVRPAGLDFR<br>AGSLGVPAGRPVSVRDLGLIAAMNRPWVSVRRRPRVAVLATG<br>DEVVMPGDPLGPSQIVSSNGLALCALVEACGGSAINLGIAAD<br>SAESLQRLAAGAAGADLLVTTGGASVGEHDLIRSVLGEAGLE<br>LDFWKIAMRPGKPLMFGRLKDTPMLGLPGNPVSSLVCGLLFL<br>RPMLDRLLGLQRPAHPLEPALLGADLGANDRRQDYLRASLET<br>DADGRRVATPFGRQDSSMLATLTQADALIVRPPHAPALAAGQ<br>VVALLRFPAGLGSIDVRQATRWEAAGPLRASNKGPDDMLTRK<br>QHELLLFLHAHLGEHGVSPSFDEMKEALGLKSKSGIHRLITG<br>LEERGFIRRLPHRARAIEVLRLPEDMAGKSGFAPNVIEGGRR<br>AGLAGARVATDSEAVSLPLYGRIAAGTPIEALRDHSNYVDVP<br>ADLLSRGEHYALQVEGDSMVEAGILDGDTVVIERSDQAENGA<br>IVVALVDDAEVTLKRFRRRGGAIALEPANRNYEPRLFPPDRV<br>KVQGRLIGLLRRY	**contaminated sequence**<br><br>**correct assignment:**<br>*Kiloniellales bacterium*
Tamarix ramosissima / Pucker *et al.*, 2024	DYLKQVLDIDLHAQIHFGRVFMKPGLPTTFATLDIDGVRKII<br>FALPGNPVSAVVTCNLFVVPALRKMQGILDPRPTIIKARLSC<br>DVKLDPRPEYHRCILTWHHQEPLPWAQSTGNQMSSRLMSMRS<br>ANGLLMLPPKTEQYVELHKGEVVDVMVIGWL	**contaminated sequence**<br><br>**correct assignment:**<br>*Myotis davidii*
Persea americana / O'Leary *et al.*, 2024	MMARPLAGVRHNTLVVTLPGSPKGAVENLQAIIKLLPHACQQ<br>AAGSNSRTLHAGGVAQLEKDAGVSSGSHSHNHDHKHHHGHSH<br>GHSDKHTHGHAVPRAHTTAAERLASNDPTAGPTRRYRESPYP<br>MLSVKDALDVIANNTPKPVAYRRPVDEDLVGHVLAEDVPAKE<br>SVPAFRASIVDGYAIIASKHVMVPSTKGIFPVVSISHAKAGS<br>VEKLEIGEVARITTGAPLPPGATSVVMVEDTVLRKSTDDGKE<br>EAEIEILTDAIEPGENVREVGSDVTAGDIILRKGEGVSATGG<br>ELGLLASVGTDTVLAYRKPRVGVLSTGDEIVPHNRQGALQLG<br>EVRDTNRPTLLTSIRAQGFDAVDLGIASDAPGALETTLRNAM<br>REVDVIVTSGGVSMGELDLLKPTIERQLGGTIHFGRVSMKPG<br>KPTTFATIPFKENDGQDTKRLIFSLPGNPASAVVTFNLFVLP<br>ALHQHSGVEPAGLPKIKVVLEQDVRCDEKRDEYHRVVIIAKG<br>DGRLYASSTGGQRSSRIGSFKSANGLLCLPAKNGSIKKGEVC<br>DALLMARLLGEA	**contaminated sequence**<br><br>**correct assignment:**<br>*Peltaster fructicola*
Anaerolineae bacterium / O'Leary *et al.*, 2024	MKHSPNSPILLNSSLHVDEARKAITNLVSELQQESSILNDPA<br>DIETVSLDHAINRVLAQDLLSPIDVPAADNSAMDGYAFDGKC<br>LSQAGSEVTLNIVGTALAGKPFEGEIGQGECLKIMTGALMPA<br>DCDTVIPQEFTTSASAESICFPSNQLKAGENRRLRGEDLQKD<br>KAAISAGRLLRPSDLGLAASLGTSHLQVRRKLRVAILSSGDE<br>LRSLGQPLDPGSIYDSNRYSLNGMLNRLNIDIIDCGIVRDNP<br>DSLKDAFIAAASKADVLISSGGVSVGEADFTKQVMLELGDVG<br>FWKIAMRPGRPMAFGTLKPVPSKSPARKTLFFGLPGNPVAVM<br>VTFYQFVRSALLQLGGVTQADLPLVQAISENAIRKKPGRTEF<br>QRAILGRNVDGKPSVRITGSQGAGILRSMSEANCFVILRHDQ<br>GNVAPGELVDIALFEGLL	**contaminated sequence**<br><br>**correct assignment:**<br>*Polynucleobacter sp.*
Tamarix ramosissima / Pucker *et al.*, 2024	RAMLLSAAVQQNCKIIDLGIARDDEEELERIFNKAFDAGVDI<br>ILTSGGVSMGDRDFVKPLLEKRGKVYFSKVCMKPGKPITFAE<br>FNLKPAEDTSANKVLAFGLPGNPVSCLVCFHLFVVPAIRHLS<br>GWANAHSLRVQARLQWPIRADPVRPEFHRAIIKWKLNDGSGI<br>PGFVAESTGHQMSSRLLSMKSANALLELPATGSVIPAGTSLA<br>AILISDLSGTPASENSLSDDKVFSIQECVKPTATDVLSTTSV<br>RVAILTVSDTVASGTGPDRR	**contaminated sequence**<br><br>**correct assignment:**<br>*Populus deltoides*

Originally identified in Species / Database	Sequence	Reason for elimination
Tamarix ramosissima /<br>Pucker *et al.*, 2024	LVRHDAERALHEGEIRDSNRPALISCLMAWGIETVDLGIVSD<br>SSKDLETVLRDALRGTMDHPPVDVIITTGGVSMGERDLLKPT<br>IERLLGGTIHFGRVAMKPGKPTTFATIPRKTSNNPRKQVAIF<br>ALPGNPASALVTMHLFVLPALHKLMGFSHPDGTEDRPSRGLP<br>RVRAVLAHPIPRDPKRTEYHRAVVTASRDGRLRASSTGLEGV<br>GQRSSRVASMAKANALLVLPPGVESLPEGELVEALMMGQIVP<br>GN	**contaminated sequence**<br><br>**correct assignment:** *Thermomyces dupontii*
Nosema bombycis /<br>O'Leary *et al.*, 2024	MQEQAEQTDEGIRFLAPVKNGQNIRRLGEDIAHGAVVFPAGT<br>RLTAAELPVIASLGIAEVEVVRKVRVAVFSTGDELQLPGQPL<br>ADGQIYDTNRLAVHLMLQELGCEVINLGIIPDDPAKLREAFI<br>QADQQADVVISSGGVSVGEADYTKAILEELGEIGFWKLAIKP<br>GKPFAFGKLNHSWFCGLPGNPVSATLTFYQLVQPLLAKLSGN<br>VGQAQPMRLRVRAASGLKKSPGRLDFQRGVLQRGPDGELVVS<br>STGHQGSHIFSSFSLGNCFIVLERERGNVEAGEWVEVEPFNH<br>LFGGL	**contaminated sequence**<br><br>**correct assignment:** *Klebsiella michiganensis*
Apophysomyces sp. BC1034 /<br>O'Leary *et al.*, 2024	MSTPNDTAPVACTSHAAPGASTDVPRPPPLSTHEALARVLAA<br>ALPLCAQPEAIEQVPTLDALNRVLAADIRSALDVPPADISAM<br>DGYAVRAADVVAGRPMRVSQRIPAGHPAAGVLEAGTAARIFT<br>GAPLPAGADTVVMQEQARVQSDTVVFDAAPPAGDWINRRGSD<br>IGKDAVILPAGTRLTPQALGLAASVGCAVLPVARRPRVAIFF<br>TGDELTMPGEPLREGAIYNSSRFTLGSLLAALGCDVTDLGIV<br>PDRFDATRDALRRAALEHDLILTSGGVSVGDEDHVRAAVQAE<br>GTLDRWQIAMKPGKPLAFGTVRRALGGESHDADTGTARDTAF<br>FIGLPGNPVSSFVTFVLFVRPFILRLAGAARVEPQALRMRAD<br>FTQKKADRRNEFLRARINDGGGLDLFPNQSSAVLTSTVWGDG<br>LIDNPPGHPIQAGETVRFLPFSALLAVREAVGTAAETVDVPD<br>GVATLGDVRTWLRSRGGAWADALADTRALRMACDHVMTGPST<br>QLTDGCEVAFFPPVTGG	**contaminated sequence**<br><br>**correct assignment:** *Mycetohabitans sp. B8*
Apophysomyces sp. BC1034 /<br>O'Leary *et al.*, 2024	MTSLQTIASCIADYDPNALPVSAARAIVRQWATPVATVERLA<br>LREALDRVLADDVVSPLDVPAHDNSAMDGYAFDGTALERGGT<br>IRLRVAGTALAGRPHDARVRTGDCIRVMTGAMLPPDCDTVVP<br>QEQVEIDVNGSIQFPATALTRGANRRRAGEDLRAGHAALLAG<br>RTIRASDLGLLASLGIAEVPVRRRLRAAFFSTGDELRSLGQP<br>LEPGCVYDSNRYTLYGMLRRLNLDVIDLGVVPDNRRALETTL<br>RNAAATADVVLSSGGVSVGDADYTRELMDTLGDVAFWRVAMR<br>PGRPFAFGRIGSGAHASESRDALYFGLPGNPVAAMVAFYQIV<br>RDALIAMTGALPHPAPLVRASALDAIGKRPGRTEYPRGIARR<br>RDDGHWEVTLTGAQGSGILRSMSDANCFVILDHHRGPVAVGD<br>TGRAATFANARRRFDMKKQISYIAPGQTAKALILVYLTFSVP<br>IMLLGVLVALIRYGSVELSTVFSALILNALLGFVLLWIACRA<br>YNWVASRFGGIEIVLSDASEEA	**contaminated sequence**<br><br>**correct assignment:** *Mycetohabitans*
Astraeus_odoratus_KAG6335354.1 / O'Leary *et al.*, 2024	MGAFVVTPQEPTMDFTAGLMPLETALSQMLDRISPLHDVETL<br>PLVRCFGRIAARDIVSPMNVPGFDNSAMDGYAVRLADLQTGN<br>ALPVAGKAFAGQPFGSEWPAGTCVRIMTGAPVPQGCDAVVMQ<br>EETEQTDDGVRFTANVKAGQNIRRTGEDITLGATVFAAGQKL<br>TVGELPVLASLGIAEVDVVRKVRVAVFSTGDELQLPGQPLQD<br>GQIYDTNRLAVHLMLEQLGCEVINLGIIPDDPEKLRAAFIEA<br>DKSADVVISSGGVSVGEADYTKTLLEELGEIAFWKLAIKPGK<br>PFAFGKLPHSWFCGLPGNPVSAALTFYQLAIPLLAKLSGNKA<br>SPLPERLRVRAATRLKKSPGRLDFQRGILARNADAPSARATA<br>LSCWSVSAATWKPANGLRLSALTTCSEADMTVELSDQEMMRY<br>NRQIVLRGFDFEGQEALKAAKVLVVGLGGLGCAAAQYLAAAG<br>VGRMTLLDFDTVSVSNLQRQTLHSDATVGQPKVDSARTALAR<br>INPNVQFTLIDAMLDDDALFAQIAQHDLVLDCTDNVAVRNQL<br>NAGCFAHKTPLVSGAAIRMEGQISVFTYADGEPCYRCLSRLF<br>GENALTCVEAGVMAPLVGVIGSLQAMEAIKVLAHYGTPAAGK<br>IVMYDAMTCQFREMKLMRNPGCEVCGV	**contaminated sequence**<br><br>**correct assignment:** *Enterobacter*

**Originally identified in Species / Database**	**Sequence**	**Reason for elimination**
Capitella_teleta_ELT99908.1 / O'Leary *et al.*, 2024	GNNIRKAGEDILEGSQVFTPGRKVRPQDIGLLASLGIANVTV<br>YQKVKVAVFSTGDELKLPGEPLRHGDIYDSNRFVIKAMLKKM<br>EIDIIDLGKIPDDKEQLRQAFLRADREADAVISSGGVSVGDA<br>DYTKEILDELGETGFWKLAIKPGKPFAFGQLPNSVFFGLPGN<br>PVSATVTFQQLAAPALRHMMNQQAEEKVELTLATKTRLKKRP<br>GRRDFQRGKLVYSNSGELQVLSTGNQGSGVMSTMSQSDCYIV<br>LAEEDGDKQAGDLVKVQLFDELLK	**contaminated sequence**<br><br>**correct assignment:** *Endozoicomonas atrinae*
Rhodothermus_profundi_WP_072714208.1 / O'Leary *et al.*, 2024	MKIILLTIGDELLTGTTVNTNAAWLGAELTAHGFTVVRAETL<br>RDDPHAICQALQRARAEADVVIVCGGLGPTHDDRTREALADC<br>LNRPLQMHAALLEQIKAYFTRRGRHMPERNQVQALVPEGFTP<br>IPNPLGTAPGLWLQDASGIVVVLPGVPHELQGLMREAVLPRL<br>QQLPGRPAILQRTLVTAGIGESELQQRLQGVESLLDEAVQLA<br>YLPSPYGVRLRLTARAASREAAQQRLSELVAFIQKRIQPYLV<br>SLSGETLEVVVGKLLRRLGATVAVAESCTGGHLADCITNVSG<br>ASTYFRGGVVAYDNAVKVEVLGVAPELLAREGAVSEAVAIQM<br>ARGVRKRLGAQVALATTGIAGPTGGTPDKPVGTVWIGLADDQ<br>VAFAQRYFLPDDRKRFKQRATAAALDLLRRHLLQQKVPRAAI<br>FHR	Similarity to G-domain
Anaeromyxobacter_oryzae_WP_248360219.1 / O'Leary *et al.*, 2024	MIVEILSTGDELLTGQVVDTNSTWLMDRLWDLGVMVRRKTLV<br>ADDRADLVAAIRETAARAELVVMSGGMGPTEDDLTAECVAAV<br>LGVPLELHESSLRVIEERFRKFGRTMTPNNRKQAMFPRGAEV<br>IPNRFGTAPGFAVKVARGEVVCLPGVPLEFKGLADEWVLPRL<br>AARLGDVPAARVLKLFGVPESHADDAMRPVMDDPANAGVRWG<br>YRAHWPEVHVKWTVPGPDAAARADRIRDAVRAIFGEAVWGEA<br>KEELPELVVARLAARGERVALAESCTGGLLAELVTRVPGASN<br>VIDLGVVAYANAMKERLLGVPAGVLAVEGAVSEPVARALAEG<br>ARRVGGAAWGVGITGIAGPTGGTPEKPVGTVHVALASAAGTV<br>HVERQYRGDRERIRRQAAYEALNLLRLALR	Similarity to G-domain
Holophaga_foetida_WP_005034889.1 / O'Leary *et al.*, 2024	MRIECIAVGSELLSTGRLDTNSVWITERLGRLGLSIHRKTAI<br>GDEPGDLRALFLEAIQRSELVICTGGLGPTFDDLTKETWAEV<br>FGAELVEDPQVRRDILDFYAARNRVPPESNFKQALVPVGARI<br>LRNPFGTAPALYWESPQGYPGRRVILLPGVPLEMKQIWEGQI<br>EALLAPLAQASVHTLRMVVGSVPESTLDERTRALREQHGALE<br>WTILAGISHVELVARGADPALLEAARKGFEGELGEDLVCVGE<br>GSIESTVLDLLQARGETLGLAESVTGGFIATRLVAVPGASQA<br>FLGDVVTYSARAKVQLAGVPERVIQVHGTVSEATTRAMAEGI<br>RDRLGATWGLATTGNAGPSQDAQGPAAVGTIHIALAGPAGTQ<br>TIQYSLPGFRSDIQSRAAAWAMDFLRRRLV	Similarity to G-domain
Nitrospina_gracillis_WP_042252643.1 / O'Leary *et al.*, 2024	MKNKYDIPQAEIVAVGNELLNGLVSDTNSTFICGQLRMHGLQ<br>VGRISVVGDDADAIRSALDQALSRVDLVIVTGGLGATHDDIT<br>KDVLADYFGTPLVRDPKVEEMIRVFFEKRQRPVPDAALRQAE<br>VPKDGRPLYNDQGTAPGLMFERGEQRVYVLPGVPREAEHLTR<br>QYILPDVAPAGNLCLQQRMLWTTGLVESALWEMFGPVDELEN<br>LVQVASLPSHLGVRIHLTAYGENVEETSAKIEQAETLLEKVL<br>SSYIYARDEQTMESVLGQLLVDRGETVAVAESCTGGLIGHRL<br>TNIPGSSRYFLQGWLTYSNEAKVKSLGVDAALVERHGAVSEE<br>VARAMAEGARQCAGTDWAVSVTGIAGPDGGTATKPVGLTYIA<br>VAGKTLTSCQKFVFPQDRLRNKERAAQAALNLLRLHLIGLK	Similarity to G-domain
