# Supplementary material for: Phylogenetic based dissection of eukaryotic Mo-insertase functionality: From mechanism to complex assembly
Source: PLoS One. 2026 Jun 12;21(6):e0350191. doi: 10.1371/journal.pone.0350191 (PMC13262936; doi:10.1371/journal.pone.0350191)
Supplement: S5 Data File — Bootstrap values are shown (pdf format). (PDF) [file pone.0350191.s017.pdf]

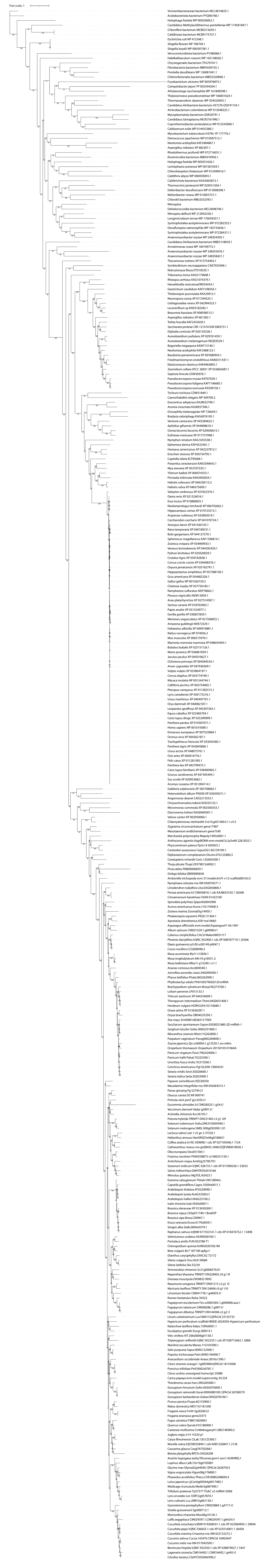
Tree scale: 1
