# Supplementary figures and images for: Phylogenetic based dissection of eukaryotic Mo-insertase functionality: From mechanism to complex assembly
Source: PLoS One. 2026 Jun 12;21(6):e0350191. doi: 10.1371/journal.pone.0350191 (PMC13262936; doi:10.1371/journal.pone.0350191)

MOCS2B

Tree scale: 1

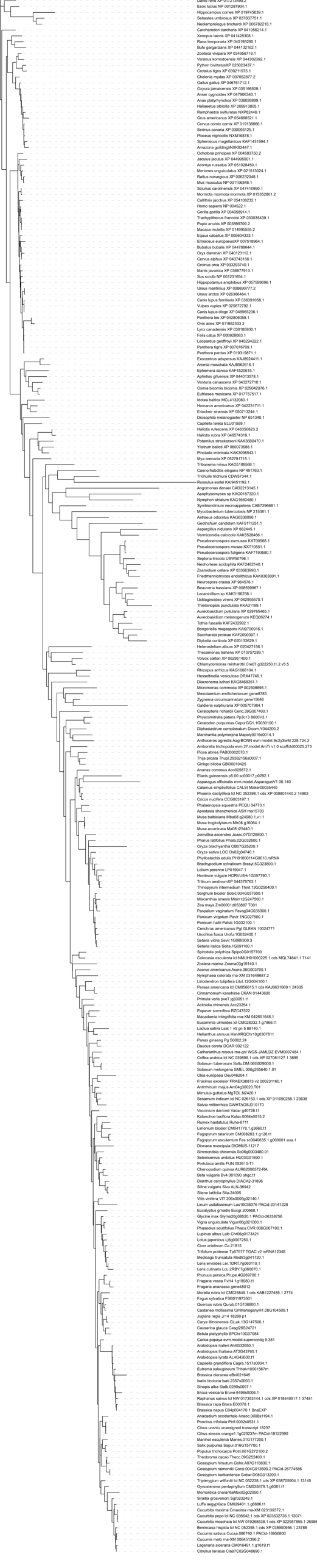

Supplement: S7 Data File — (PDF) [file pone.0350191.s019.pdf]

ADH

Tree scale: 1

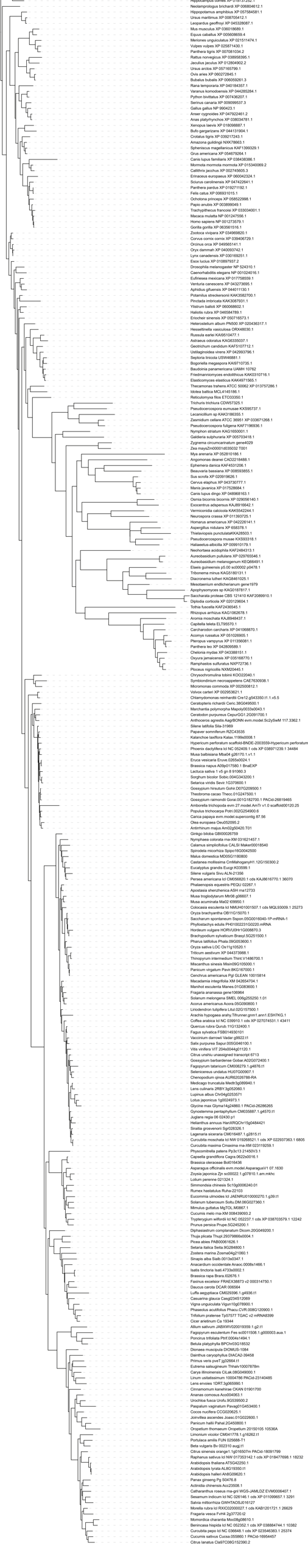

Supplement: S8 Data File — (PDF) [file pone.0350191.s020.pdf]
